# A new species of *Roupala* (Proteaceae) from Central Brazil

**DOI:** 10.3897/phytokeys.13.2836

**Published:** 2012-05-28

**Authors:** Ghillean T. Prance

**Affiliations:** 1 Royal Botanic Gardens, Kew, Richmond, Surrey, TW9 3AB, UK

**Keywords:** Proteaceae, *Roupala*, Brazil, Atlantic rainforest

## Abstract

A new species of *Roupala*, *Roupala gertii* from the endangered Atlantic coastal forests of Brazil is described and illustrated.

## Introduction

The Neotropical representatives of the Proteaceae were monographed in 2007 (Prance et al. 2007) building on the ealier work of Sleumer (1954). The monograph included thirty-three species of *Roupala*. Since that time a collection has come to my attention that does not fit into any of the previously described taxa. This is presented here as *Roupala gertii*. It is currently only known from a single collection from the rather poorly collected Atlantic coastal forest of the State of Espírito Santo, Brazil a region from where novelties are to be expected.

## Description

### Roupala gertii

Prance sp. nov.

urn:lsid:ipni.org:names:77119674-1

http://species-id.net/wiki/Roupala_gertii

Fig. 1

#### Latin.

Ab R. paulensis foliis minoribus acuminis 3-8 mm longis, petiolis 8-20 mm, rachidibus inflorescentiis tenuibus differt.

#### Type.

Brazil. Espírito Santo, BR-262 road near junction to Laranja da Terra, Iúna Municpality, 7 Nov 1993 (fl), *G & M Hatchbach & J. M. Silva 59702* (holotype, MBM Curitiba; isotype, K).

Tree 6 m tall, young branches appressed-tomentose, not conspicuously lenticellate. Leaves simple, lamina chartaceous, drying reddish-brown, ovate, 4.5–8.5 × 2–4.4 cm, length:breadth ratio 1–2.2–2.7, semicraspedodromous: ferrugineous-tomentose beneath, glabrous above except along midrib, the margins distinctly revolute, entire or slightly serrate, cuneate at base, equal or slightly unequal; apex acuminate, the acumen 3–8 mm long; primary veins 5–6 pairs, prominulous beneath, slightly prominulous above; midrib reaching apex of lamina, prominent beneath, plane above, tomentose, glabrescent; petioles 8–20 mm, tomentose when young, terete, lamina slightly confluent onto petiole for up to 5 mm. Inflorescence terminal or axillary; peduncle 3–10 mm × 1 mm thick; rachis 4.5–8.5 × 1 mm, ferrugineous-tomentose; bracts few, 1 × 1 mm, tomentose; pedicels 1.5–2 mm, ferrugineous-tomentose, free to base; flowers borne solitary or in pairs, 4–5 mm long, exterior ferrugineous-tomentose.; tepals 4, reflexed and twisted when open; filaments adnate to upper half of tepals; anthers 1–1.2 mm long; nectary lobes 4, well separated, conspicuous; ovary erect, densely tomentose; style clavate. 3–4 mm long. Fruit not seen.

This species clearly belongs to the genus *Roupala* on account of its four hypogynous glands and bilocular ovary with pendulous ovules. It is near to *Roupala paulensis* Sleumer but differs in the thinner smaller leaves that dry brown above rather than green. The plants is far less robust, the petioles are shorter, the leaves more acuminate and the inflorescence rachis much thinner. It is a pleasure to name this species for Dr Gert Hatschbach who has done so much make the flora of south and central Brazil so much better known and who made the first collection of this species.

**Figure 1.**
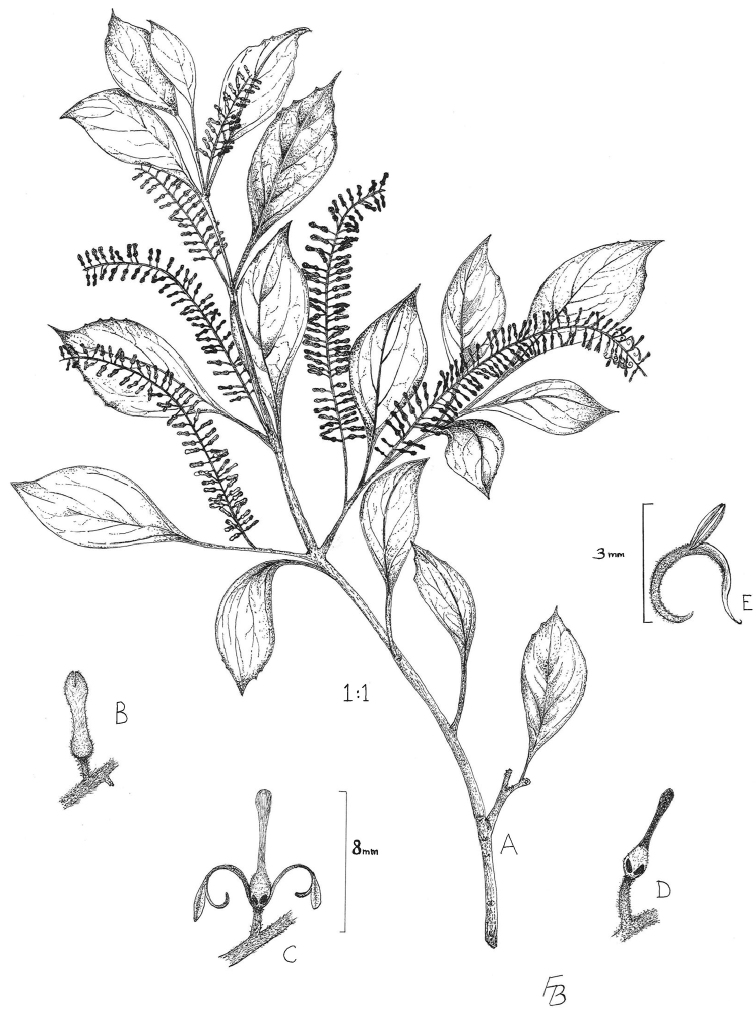
*Roupala gertii* Prance **A** habit **B** flower bud **C** open flower with two tepals removed **D** Ovary and style showing two of the nectary lobes **E** tepal and stamen. (drawn by Flora Bamford)

## Supplementary Material

XML Treatment for Roupala gertii

## References

[B1] Prance GT Plana V Edwards KS Pennington RT (2007) Proteaceae. Flora Neotropica Monograph 100: 1-218

[B2] Sleumer H (1954). Proteaceae americanae. Botanische Jahrbuecher fuer Systematik 76: 139-211

